# Brazilin isolated from *Caesalpinia sappan* L. inhibits rheumatoid arthritis activity in a type-II collagen induced arthritis mouse model

**DOI:** 10.1186/s12906-015-0648-x

**Published:** 2015-04-22

**Authors:** Eui-Gil Jung, Kook-Il Han, Seon Gu Hwang, Hyun-Jung Kwon, Bharat Bhusan Patnaik, Yong Hyun Kim, Man-Deuk Han

**Affiliations:** Department of Life Science and Biotechnology, Soonchunhyang University, Asan, Chungnam, 336-745 Republic of Korea; Department of Dental hygiene, Gimcheon University, 214 Daehakro, Gimcheon City Gyungbuk, 740-704 Korea; Division of Plant Biotechnology, College of Agriculture and Life Science, Chonnam National University, Gwangju, 500-757 Republic of Korea; School of Biotechnology, Trident Academy of Creative Technology (TACT), Bhubaneswar, 751007 Odisha India

**Keywords:** *Caesalpinia sappan*, Rheumatoid arthritis, Collagen-induced arthritis, Brazilin, Pro-inflammatory cytokines

## Abstract

**Background:**

*Caesalpinia sappan* L. extracts exhibit great therapeutic potential, and have been shown to have analgesic and anti-inflammatory properties. This study aimed to understand the anti-rheumatoid activity of brazilin that was isolated from ethyl acetate extract of *C. sappan* L. The evaluations were conducted in mice with type-II collagen-induced arthritis (CIA).

**Methods:**

Brazilin was purified via preparative HPLC and identified by mass spectrometry and ^1^H/^13^C NMR analysis. DBA/1J mice were divided into four groups (*n* = 10). Three groups of mice received intradermal injections of inducer bovine type-II collagen (BTIIC; 2 mg/ml in 0.05 ml acetic acid) and 0.1 ml of booster complete Freund’s adjuvant (CFA). A second injection of BTIIC with booster incomplete Freund’s adjuvant (ICFA) was given subsequently after 21 days. On 22nd day, purified brazilin (10 mg/kg body weight) or the disease-modifying anti-rheumatic drug methotrexate (3 mg/kg body weight) was administered intraperitoneally daily or every three days for 21 days, respectively to two groups of mice. At the 42nd day, mice sera were collected, and the levels of pro-inflammatory cytokines and stress enzyme markers in serum were measured using standard immunoassay methods. The microstructure and morphometric analyses of the bones were assessed using high-resolution microfocal computed tomography.

**Results:**

Brazilin isolated from *C. sappan* reduced the arthritis index score and the extent of acute inflammatory paw edema in CIA-mice. The bone mineral density was significantly (*p* < 0.05) lower in only-CIA mice, and appeared to increase commensurate with methotrexate and brazilin administration. Brazilin prevented joint destruction, surface erosion, and enhanced bone formation as revealed by microstructural examinations. Brazilin markedly attenuated mouse CIA and reduced the serum levels of inflammatory cytokines including TNF-α, IL-1β, and IL-6.

**Conclusions:**

Brazilin purified from *C. sappan* L. shows protective efficacy in CIA mouse, and may be useful to treat chronic inflammatory disorders including rheumatoid arthritis.

## Background

Rheumatoid arthritis (RA) is a systemic inflammatory disease of the synovium, cartilage, and bone. RA causes persistent pain, stiffness, swelling, deformities, and loss of joint function [1,2]. Although RA is not inherited, certain human RA susceptible genes may be triggered in response to infection or environmental factors, establishing an autoimmune condition wherein the immune system begins to produce substances that attack the joints. Such substances include interleukin-1 (IL-1) and tumor necrosis factor alpha (TNF-α); these factors damage articular cartilage and bone [3]. Some reports have documented the synthesis of novel cytokines including IL-17, IL-18, and RANK ligand in the synovium, which secretes enzymes degrading proteoglycans and collagen, in turn causing bone loss [4,5].

The worldwide prevalence of RA is 0.5 - 1% [6], and RA is threefold more prevalent in females than in males. Three general classes of drugs have been recommended to treat RA; these are non-steroidal anti-inflammatory agents (NSAIDs), corticosteroids, and disease-modifying anti-rheumatic drugs (DMARDs). Although all of these drugs reduce acute inflammation and pain, they do not change the course of the disease or prevent joint destruction. Additionally, drug side-effects include increased risks of malignancy and gastrointestinal disturbances. These side-effects and the high drug costs mitigate against prolonged drug use [7].

Methotrexate (MTX) is a DMARD, and is classified pharmacologically as an anti-folic-acid because the drug antagonizes folic-acid metabolism [8,9]. MTX not only reduces pain and swelling in the joints, but also its damage and long-term disability. However, the side-effects include liver functional abnormalities, lung disorders, stomatitis, skin rashes, hematological diseases, and pancytopenia. In short, MTX must be administered only with care. To compensate for the adverse effects of MTX and other drugs, and to devise cost-efficient RA treatment strategies, attention has recently been diverted to the study of useful natural compounds derived from herbal plants [10]. Several recent studies have focused on herbal extracts or compounds that exhibit anti-arthritic effects in rats with adjuvant-induced arthritis [11-13].

*Caesalpinia sappan* L. (Leguminosae) is a traditional medicinal plant distributed in the Asian peninsula including India, Burma, Vietnam, Sri Lanka, and China. The dried heartwood of *C. sappan* L. exhibits various pharmacological effects, including anti-hyperglycemic, anti-hypercholesterolemic, anti-hepatotoxic, anti-inflammatory, and sedative activities [14-18]. The immunomodulatory, anti-inflammatory, and antioxidant activities of *C. sappan* extracts suggest its potential role in exerting antiarthritic effects.

In the present study, we extracted and characterized a major component of the dried heartwood of *C. sappan*; brazilin (7,11*b*-dihydrobenz [*b*] indeno [1,2-*d*]pyran-3,6a,9,10(6H)-tetrol). This is a natural red pigment with both anti-inflammatory and anticancer activities [17,19]. We explored whether brazilin purified from *C. sappan* could be used to treat type-II collagen-induced arthritis (CIA) in a mouse model of RA.

## Methods

### Plant materials and isolation of the compound

Dried heartwood of *C. sappan* L. was purchased from the Kyungdong Local Market, Seoul, Korea in March 2012. A voucher specimen (No. SCHB 12–015) was deposited at the Herbarium of College of Natural Science, Soonchunhyang University and authenticated by Dr. B.Y. Lee from National Institute of Biological Resources, South Korea.

Air-dried and chipped *C. sappan* (6 kg) was extracted with 95% (v/v) methanol for 72 h. The extract was filtered through a Buchner funnel fitted with Whatman No. 1 filter paper, concentrated in a rotary evaporator operating under reduced pressure, and subsequently diluted in water. This preparation was further fractionated into hexane (3 x 1,000 ml), chloroform (3 x 1,000 ml), ethyl acetate (3 x 1,000 ml), and butanol saturated with water (3 x 1,000 ml) extracts. Each extract, and the aqueous phase remaining after all extractions, was dried under reduced pressure to yield a hexane (1.13 g), a chloroform (7.05 g), an ethyl acetate (176.94 g), a butanol (8.26 g), and a water fraction (4.35 g).

The ethyl acetate soluble fraction (170 g) was purified by column chromatography (CC) on a Sephadex LH-20 matrix, and yielded five fractions (C-1 ~ C-5). Fraction C-4 (7.31 g) was further separated by CC on a Sephadex LH-20 matrix, using CHCl_3_: MeOH (20:1, v/v), to generate fractions C-4-1 ~ C-4-4 [19]. Fraction C-4-3 (3.54 g) was separated via silica gel CC, using CHCl_3_: EtOAc (7:3, 5:5, 3:7), to yield three fractions. Fraction C-4-3-2 (401 mg) was again purified via silica gel CC using CHCl_3_: EtOAc (7:3, 5:5) to yield fractions C-4-3-2-1 and C-4-3-2-2. The effective ingredient in fraction C-4-3-2-1 was confirmed to be brazilin.

### High-performance liquid chromatography (HPLC)

Brazilin was purified from *C. sappan* extract using a preparative LC-20 series HPLC system (Shimadzu Corp., Tokyo, Japan) fitted with a reverse-phase C_18_ column (UG120, 5-μm particle sizes, 4.6 I.D. x 250 mm UG120, Shiseido, Japan) with monitoring of eluate absorbance at 280 nm. The eluent system consisted of an isocratic mode of 100% (v/v) methanol, running at a flow rate of 0.5 ml/min at a column temperature of 25°C. Fraction with retention times of about 7.669 were amalgamated from repeat HPLC runs.

### Spectrometric identification of the compound

A liquid chromatography-mass spectrometry-ion trap-time of flight (LCMS-IT-TOF) mass spectrometer (Shimadzu Corporation, Tokyo, Japan) was used for mass spectrometric evaluation of the positive and negative ion mode masses and to record MS/MS spectra. The detection voltage and interface temperature were 1.60 V and 400°C respectively.

^1^H nuclear magnetic resonance (NMR) and ^13^C NMR spectra were obtained with the aid of a JNM-LA 400 NMR (Jeol Ltd., Tokyo, Japan) operating at 100 MHz, using CD_3_OD as solvent. Chemical shifts are reported in parts per million (ppm) downfield from those of an internal tetramethylsilane (TMS) standard.

### Drugs and experimental animals

Brazilin was kept in brown glass bottle and a vacuum container. The extract was stored at 4°C until further use. Brazilin and MTX were dissolved in a small volume of dimethylsulfoxide (DMSO) and its concentration was adjusted to working concentration with 0.9% saline for animal administration. The final concentration of DMSO was set to 0.1%.

Arthritis was induced in 7-week-old male DBA/1J mice (body weight 20–23 g; Central Laboratory, Animal Inc., Seoul, Korea), and were prospectively randomized into normal, control, and treatment groups. The animals were acclimated under standard laboratory conditions of 22 ± 2°C ambient temperature, relative humidity of 60 ± 5% under a daily 12 h light: dark cycle. All mice had free access to tap water and a laboratory diet [crude protein NLT 20.5%, crude fat NLT 3.5%, crude fiber NMT 8.0%, crude ash NMT 8.0%, Ca NLT 0.5%, and phosphorus NLT 0.5% (all w/w)]. After 7 days of acclimatization, mice were used in experiments. The study was conducted with the consent of the Ethics Committee for the use of experimental animals as authorized by the Animal Research Center of Soonchunhyang University (Approval number: SCH13_08_03).

### Induction of CIA

DBA/1J mice were injected intradermally twice (each of 100 μg) with bovine type-II collagen (Central Lab, Seoul, Korea) dissolved overnight at 4°C in 0.05 M acetic acid (2 mg/ml), and subsequently emulsified in an equal volume of complete Freund’s adjuvant (CFA, Sigma-Aldrich, St. Louis, MO, USA) at day 0 (primary immunization), and incomplete Freund’s adjuvant (ICFA, Sigma-Aldrich, St. Louis, MO, USA) at day 21 (2nd booster injection) [20]. The animals were randomly divided into four groups of approximately equal mean body weight (*n* = 10 per group). It included normal group without immunization or CIA, control group with saline injection after CIA, and experimental groups with intraperitoneal injections of anti-rheumatoid drug, methotrexate (3 mg/kg of mouse body weight; 0.2 mL) every 3rd day and brazilin (10 mg/kg of mouse body weight; 0.2 mL) daily from day 22 to day 42 after 2nd booster injection.

### Clinical scoring of arthritis

CIA mice were evaluated by two independent observers three-to-four times in a week, with regards to the extent of all the inflammation, erythema, edema of the periarticular tissues, and enlargement, distortion, or ankylosis of the joints. Each paw was scored on a scale of 0–4 (an arthritis index), where 0 indicated inflammation; 1 edema or erythema of one joint; 2 edema or erythema of two joints (whether of one or two digits); 3 edema or erythema of more than two joints (whether of one, two, or three digits); and 4 severe arthritis of the entire paw or all digits [21]. Each total arthritis score was the sum of the scores for all four limbs (the maximum possible score was thus 16). Each arthritis index was derived by two independent observers blinded to treatment mode. Paw thickness was measured at least three-to-four times per week, to monitor footpad swelling. The thickness of the paw was measured using a Vernier calliper (Ozaki, Tokyo, Japan) at least 3 times a week for footpad swelling. Each mouse was weighed 2 times a week.

### Microstructural bone examination using microfocal computed tomography (micro-CT)

The microstructures of the distal end of the left femur, the proximal end of the left tibia, the distal end of the left calcaneus, and the distal end of the left second metatarsal bone, were analyzed via high resolution micro-CT (SkyScan 1172, Bruker, Antwerp, Belgium). Bone mineral density (BMD) was determined by running metaphyseal scans at points 3% along the length of each bone. The slice pitch was 20 μm, and 50 slices were analyzed. The two-dimensional images thus obtained were converted to three-dimensional images, and bone microstructure was evaluated. The parameters recorded were: bone volume (in mm^3^) relative to tissue volume (in mm^3^) (expressed as a percentage); bone surface (in mm^2^) relative to bone volume (in mm^3^) (expressed per mm); trabecular thickness (in mm); trabecular number (in mm^−1^), and fractal dimension.

### Serum biochemical analysis

The serum levels of the proinflammatory cytokines TNF-α, IL-1β, and IL-6 were measured using mouse immunoassay kits (IL-1β: ML800C; TNF-α: MTA00B; and IL-6: M6000B; R&D Systems, Minneapolis, MN) according to the manufacturer’s instructions. The levels of enzyme markers of stress, including aspartate aminotransferase (AST), alanine aminotransferase (ALT), alkaline phosphatase (ALP), and creatinine levels were measured on a Synchron LX-20 Clinical System (Beckman Coulter Inc., Brea, CA).

### Statistical analysis

Statistical analysis featured one-way analysis of variance. All experimental values are expressed as means ± standard errors. Between groups comparisons were performed using the post-hoc Tukey test. *p <* 0.05 was considered to indicate significance.

## Results

### Purification and characterization of brazilin

**Figure 1:**
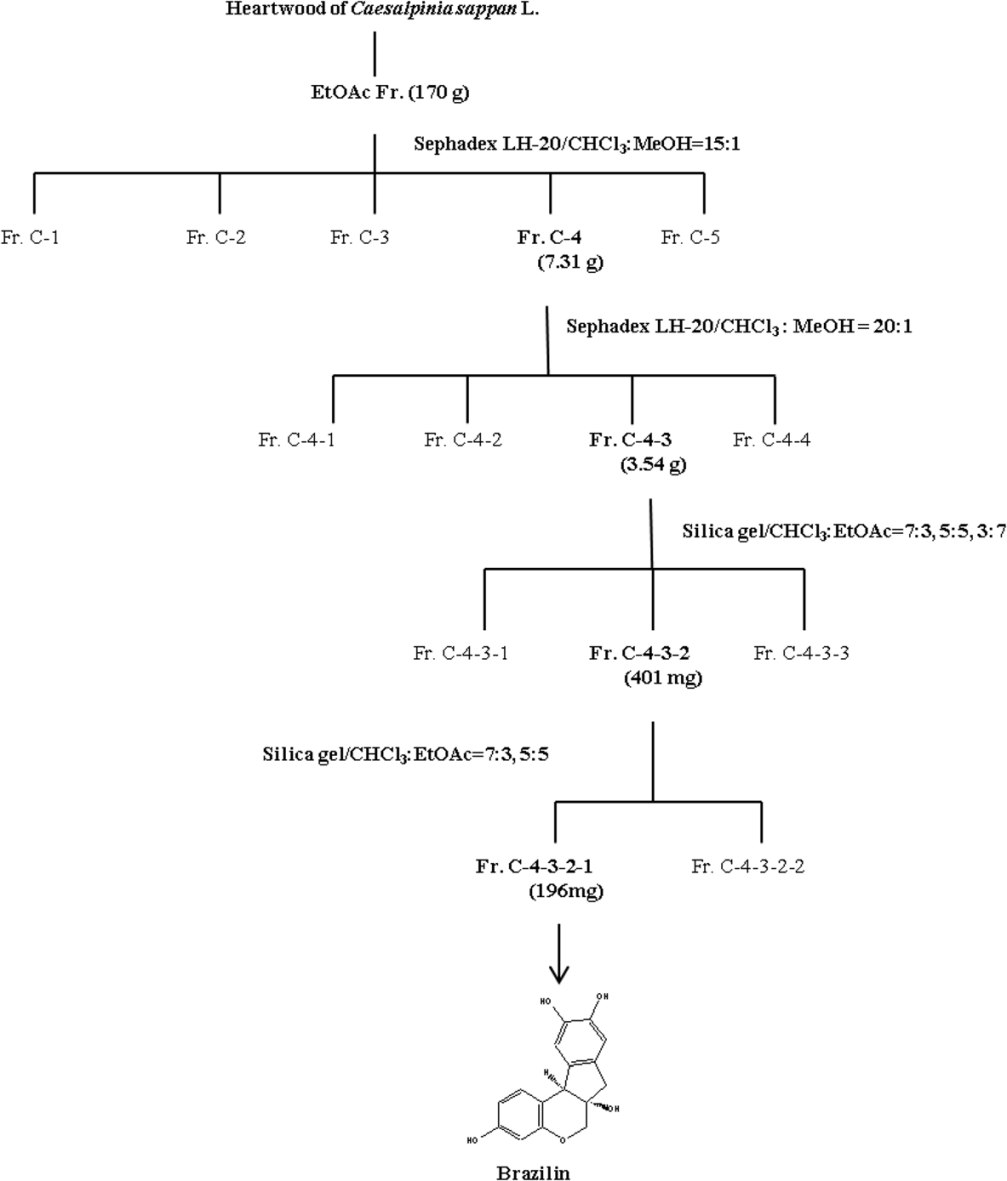
Schematic diagram illustrating the sequential purification of brazilin (7,11*b*-dihydrobenz [*b*] indeno [1,2-*d*] pyran-3,6a,9,10(6H)-tetrol) from methanol extract of *Caesalpinia sappan* L. The chemical structure of brazilin is shown below.

**Figure 2:**
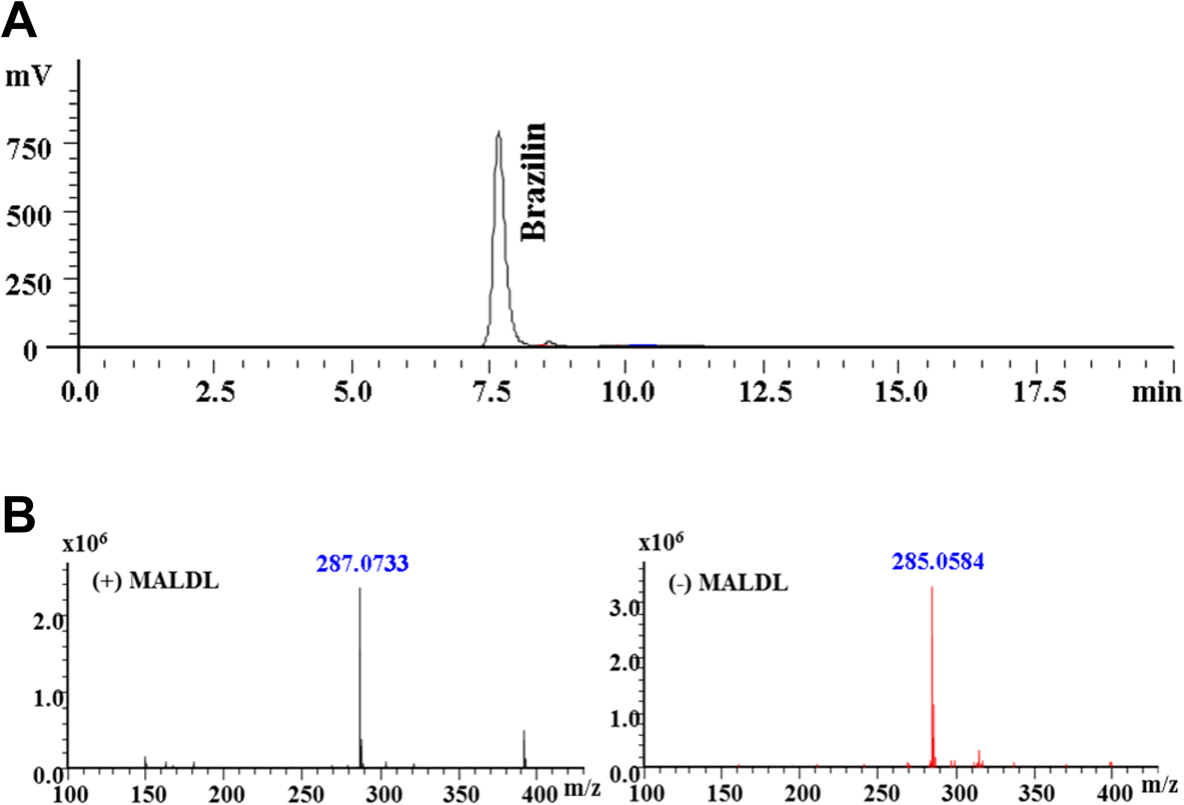
Purification and characterization of brazilin from *Caesalpinia sappan* L. extracts. **(A)** Preparative HPLC profile of purified brazilin from *C. sappan* extract. A reverse phase C18 column (4.6 x 250 mm) was used for analysis, and eluted with 100% methanol in isocratic mode with flow rate of 0.5 ml/min and detection wavelength of 280 nm. A near-homogenous peak with a retention time of 7.669 min was observed. **(B)** MALDI-TOF mass spectra of brazilin from *C. sappan* L. The optimal collision energy was 45eV in the mass range m/z 100 to m/z 450. The detection voltage and interface temperature were set to 1.60 V and 400°C. Methanol was used as the mobile phase.

The ethyl acetate extract of *C. sappan* heartwood was repeatedly extracted with CHCl_3_: MeOH and subjected to Sephadex and silica gel column chromatography to obtain a single final fraction (Figure 1), later identified to contain brazilin which exerted anti-RA effects in the mouse CIA model. Brazilin formed a single homogenous peak under preparative HPLC conditions, with a retention time of 7.669 min (Figure 2A). The purity of brazilin was determined to be above 98% according to HPLC conditions. LCMS-IT-TOF mass spectrometry yielded the elemental composition and molecular weight of brazilin which had a positive ion mass of 287.0733 m/z and a negative ion mass of 285.0584 m/z (Figure 2B).

**Figure 3:**
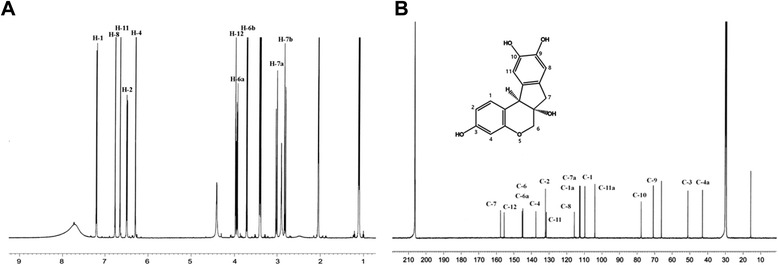
Nuclear magnetic resonance (NMR) spectra of brazilin from *Caesalpinia sappan* L. **(A)** ^1^H-NMR spectrum **(B)** ^13^C-NMR spectrum.

We used ^1^H-NMR (Figure 3A) and ^13^C-NMR (Figure 3B) to derive the chemical structure of brazilin. ^1^H-NMR data: (400 MHz, CD_3_OD)δ: 7.19 (1H, d, *J* = 5.6 Hz, H-1), 6.77 (1H, br s, H-8), 6.65 (1H, br s, H-11), 6.49 (1H, dd, *J* = 11 and 1.6 Hz, H-2), 6.30 (1H, d, *J* = 1.6 Hz, H-4), 3.98 (1H, br s, H-12), 3.93 (1H, dd, *J* = 7.2 and 0.8 Hz, H-6a), 3.71 (1H, d, *J* = 7.2, H-6b), 3.00 (1H, d, *J* = 10.4 Hz, H-7) and 2.81(1H, d, *J* = 10.4 Hz, H-7b). The ^13^C-NMR data: (100 MHz, CD_3_OD)δ 43.00 (C-4a), 51.24 (C-3), 70.92 (C-9), 77.84 (C-10), 104.08 (C-11a), 109.71 (C-1), 112.42 (C-7a), 112.74 (C-1a), 115.69 (C-8), 131.59 (C-11), 132.09 (C-2), 137.51 (C-4), 144.89 (C-6a), 145.23 (C-6), 155.64 (C-12), and 157.71 (C-7).

### Effects of brazilin on mouse CIA

**Figure 4:**
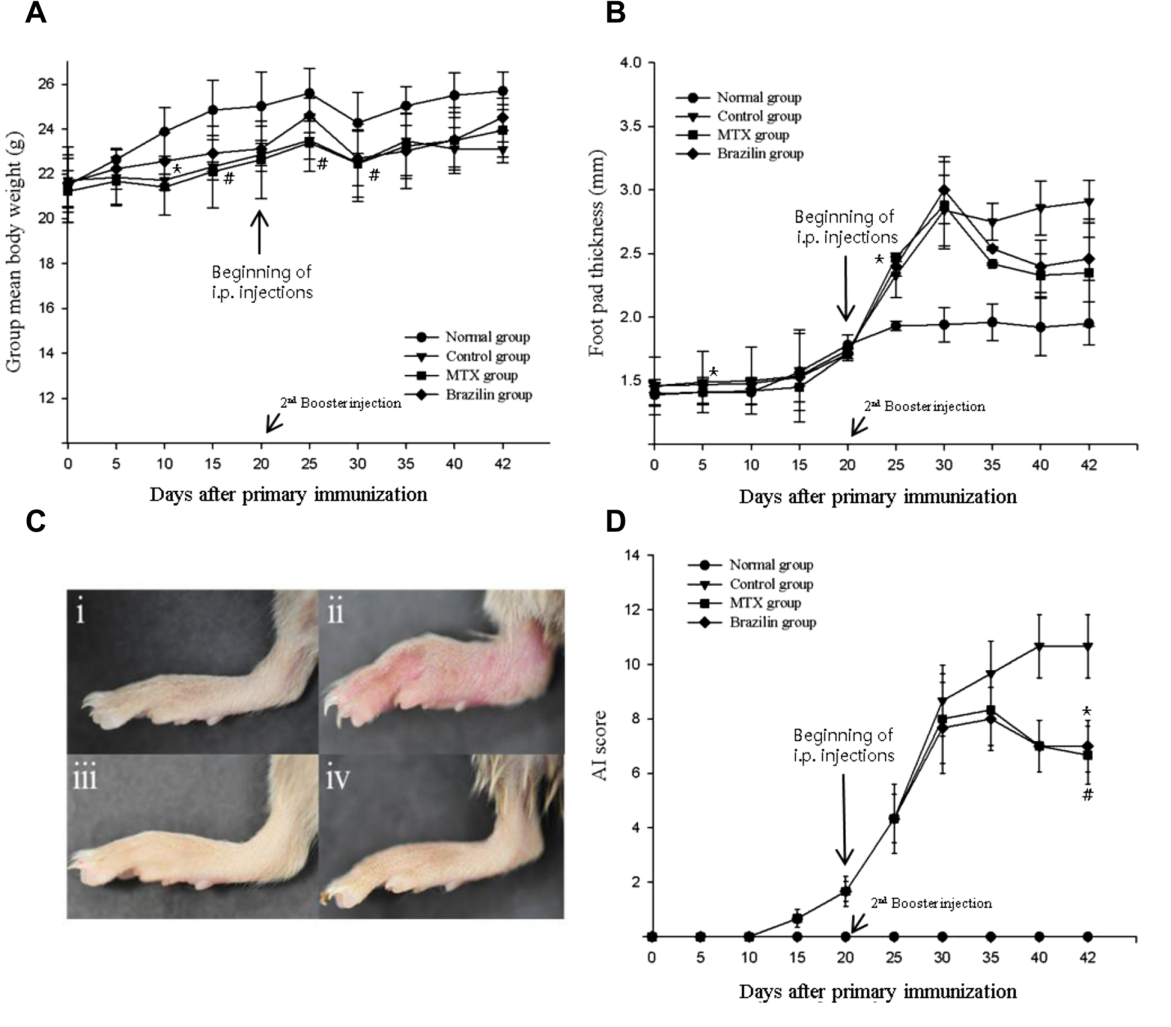
Changes in **(A)** body weight, **(B)** foot pad thickness, and **(C)** gross lesions of the hind paws of DBA/1J mice with type-II collagen induced arthritis (CIA). (C-i) normal group (CIA was not induced); (C-ii) control group (CIA was induced); (C-iii) CIA-induced mice with intraperitoneal (i.p.) injection of drug methotrexate (MTX: 3 mg/kg of body weight; injected every third day from day 22 to day 42 after the 2nd booster injection); (C-iv) CIA-induced mice with i.p. injection of brazilin (10 mg/kg of body weight; injected daily from day 22 to day 42 after the 2nd booster injection). **(D)** Arthritis index scores (means ± standard errors). *p < 0.05 (brazilin-treated compared to control group); ^#^ *p <* 0.05 (MTX treated compared to control group). Statistical analysis employed Fisher’s protected least-difference post hoc test.

Body weight decreased significantly by day 25 in mice that developed arthritis, regardless of whether such animals received brazilin or MTX (Figure 4A). No significant difference was evident among the three collagen-sensitized groups. Paw swelling was most severe (upto 3.0 mm) in the CIA-induced control group, at day 30. A steep increase in swelling was observed from day 20–30 in control group; followed by a minor decline (Figure 4B). However, marked reductions in paw swelling was evident after intraperitoneal injection of either MTX or brazilin, compared with the control group. The extent of swelling was less in MTX than brazilin-treated mice on day 42. The gross lesions in the foot of collagen-induced control group mice (Figure 4C-ii) was severe, but was lesser in MTX (Figure 4C-iii) and brazilin-treated groups (Figure 4C-iv). Arthritis scores attained their maxima by day 42 in all mouse groups (Figure 4D), and were similar at all time-points after sensitization, in mice administered either MTX or brazilin. Thus, no between-group difference was found to be significant.

**Figure 5:**
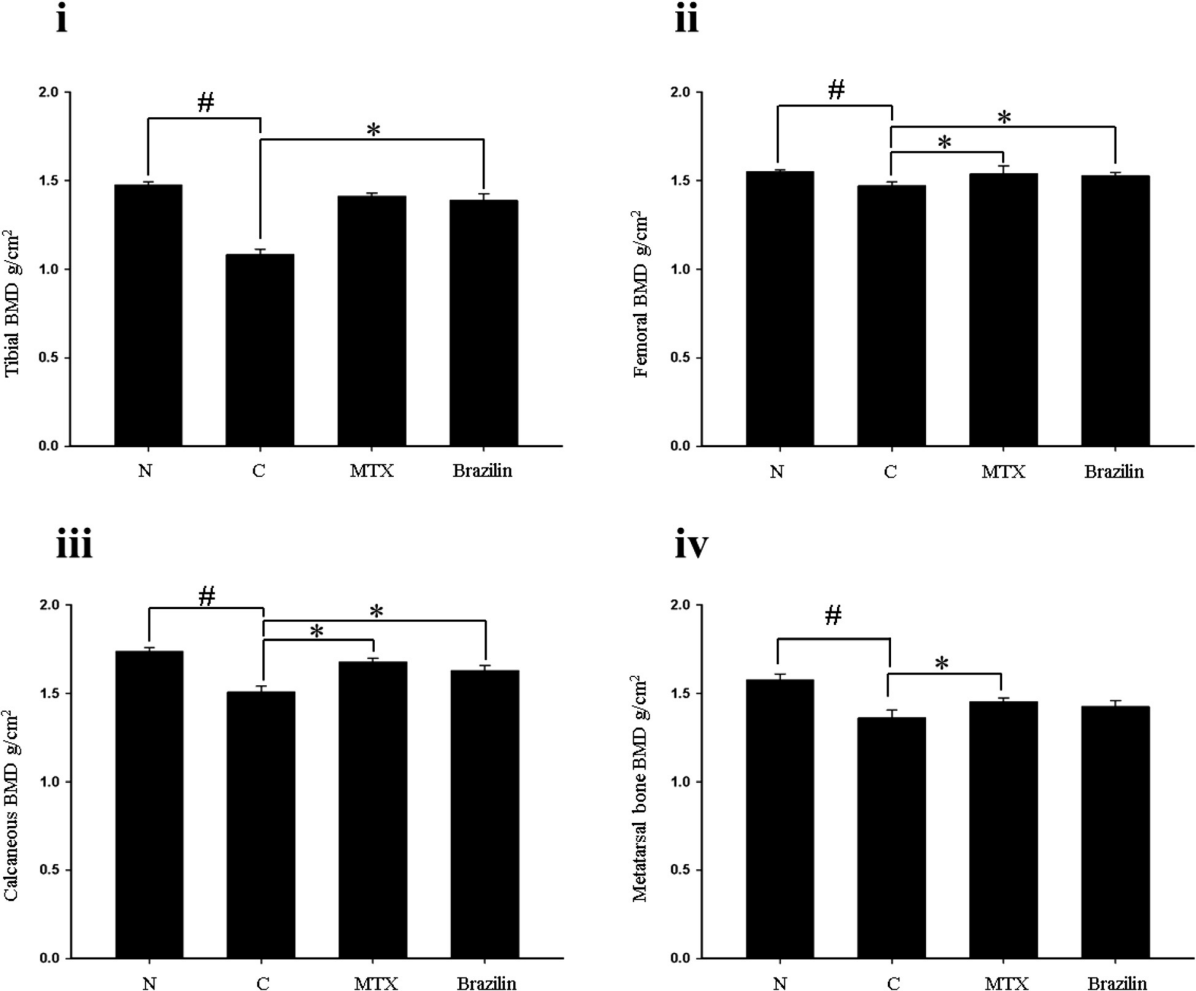
Bone mineral density (BMD) of **(i)**, the proximal part of the left tibial metaphysis; **(ii)**, the distal part of the left femoral metaphysis; **(iii)**, the distal part of the left calcaneous; and **(iv)**, the distal part of the left second metatarsal bone. Values are means ± standard errors.*p < 0.05 (versus CIA mice); ^#^ *p <* 0.05 (versus normal group. Statistical analysis employed by Fisher’s protected least difference post hoc test.

**Figure 6:**
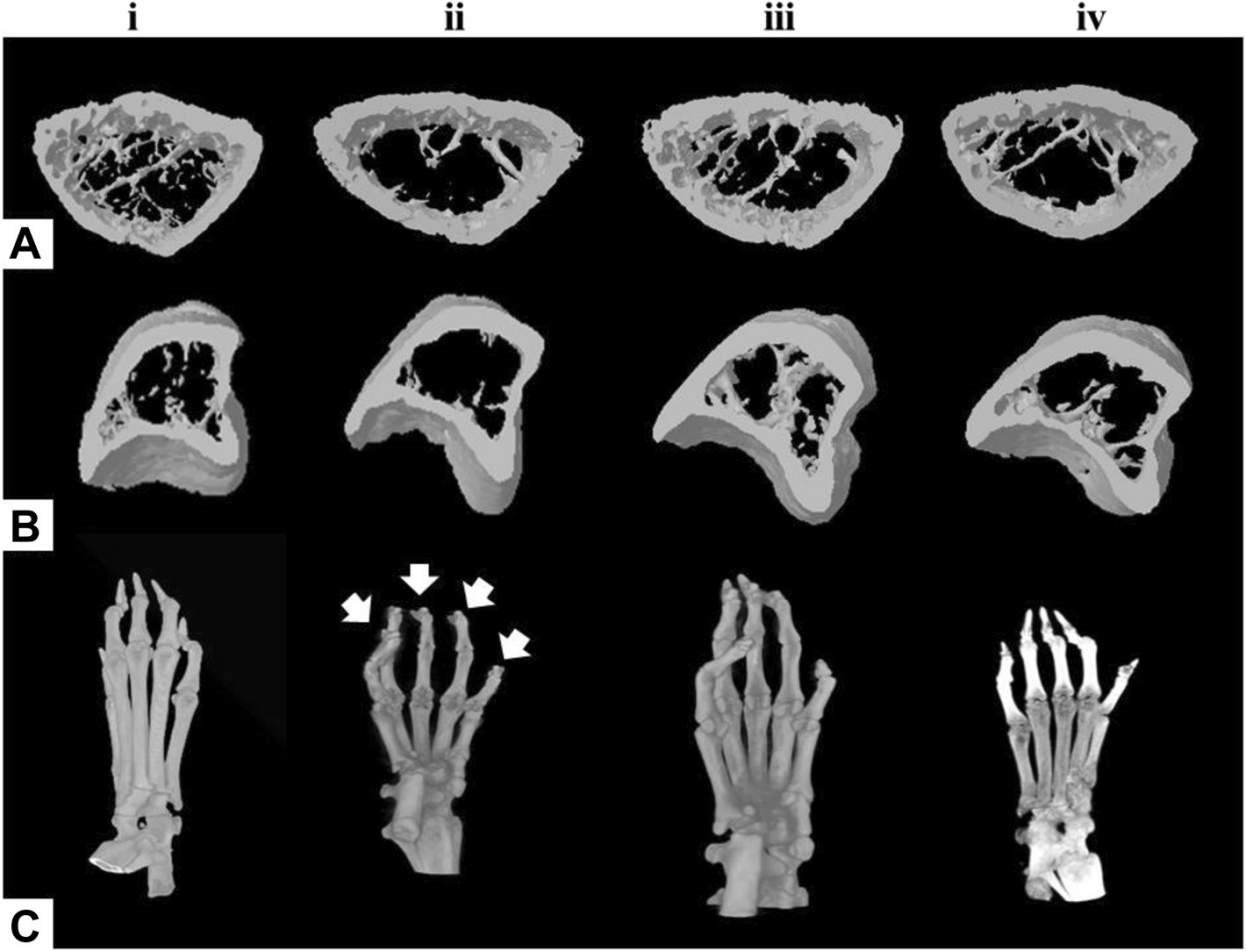
Microfocal computed tomography (micro-CT) images of DBA/1J mice with collagen-induced arthritis (CIA). **A**, the proximal part of the left tibial metaphysis; **B**, the distal part of the left femoral metaphysis; **C**, the left leg. (i) normal group (CIA was not induced); (ii) control group (CIA was induced); (iii) CIA-induced mice treated with methotrexate (MTX); (iv) CIA mice treated with brazilin.

The volumetric BMDs of CIA-induced mice were recorded (Figure 5). The BMD of the proximal part of the left tibial metaphysis (Figure 5-i), the distal part of the left femoral metaphysis (Figure 5-ii), the distal part of the left calcaneous (Figure 5-iii), and the distal part of the left second metatarsal bone (Figure 5-iv), were significantly lower in the control group than in other groups (*p <* 0.05). Administration of brazilin to CIA-induced mice significantly increased the BMD of the distal part of the left calcaneous. The decrease in BMD of the control group was not notably affected when either MTX or brazilin was administered. Micro-CT analysis of the tibial metaphysis, the femoral metaphysic, and the left leg are shown in Figure 6. The left tibial metaphysis and the distal part of the left femur of the control group were thin and distorted, compared with those of the normal group. The proximal part of the left tibial metaphysis (Figure 6A), and the distal part of the left femur (Figure 6B) were not so affected in the brazilin and MTX groups. Thus, administration of brazilin more effectively preserved trabecular architecture than did MTX.

The bone volume (BV)/tissue volume (TV) ratios were significantly lower in the control and MTX-treated mice with CIA than in the brazilin-treated group (*p <* 0.05) (Table 1). The bone surface (BS)/bone volume (BV) ratio was higher in the control group, although the trabecular thickness (Tb-Th) was significantly lower. The BS/BV ratio was significantly lower in the brazilin treated group than the normal non-CIA-induced group. The normal Tb-Th ratio was higher than those of the MTX and brazilin-treated groups (*p <* 0.05). The extent of trabecular separation (Tb.Sp) was lower in the normal group than in the groups in which CIA was induced.

**Table 1:** **Microstructure of cancellous bone at the distal part of the left femur**

**Groups (** ***n*** **= 10)**	**BV/TV (%)**	**BS/BV (mm)**	**Tb.Th (mm)**	**Tb.N (mm** ^**−1**^ **)**	**Tb.Sp (mm)**	**Fractal dimension**
**Normal**	35.02 ± 2.65	18.83 ± 3.22	0.106 ± 0.004	3.30 ± 0.23	0.20 ± 0.03	2.49 ± 0.021
**Control**	28.41 ± 1.47*	23.36 ± 3.15*	0.086 ± 0.004*	3.32 ± 0.74	0.22 ± 0.05	2.39 ± 0.071
**MTX**	33.38 ± 3.12*	19.71 ± 2.71*	0.102 ± 0.003*^#^	3.29 ± 0.23*^#^	0.20 ± 0.04	2.46 ± 0.052
**Brazilin**	32.53 ± 4.03*	20.23 ± 3.13*	0.099 ± 0.006*^#^	3.29 ± 0.21*	0.21 ± 0.02	2.41 ± 0.041

Four groups of mice (*n* = 10 each) were evaluated: control mice without collagen-induced arthritis (CIA) (normal) and CIA mice treated with saline (control group), with therapeutic administration of methotrexate (MTX: 3 mg/kg of body weight; injected every third day from day 22 to day 42 after 2nd booster injection) or brazilin (10 mg/kg of body weight; injected daily from day 22 to day 42 after the 2nd booster injection). BV - bone volume; TV - tissue volume; BS - bone surface; Tb.Th - trabecular thickness; Tb.N - trabecular number; Tb.Sp - trabecular separation; *p < 0.05 versus normal group. ^#^ *p* < 0.05 versus control group.

### Effects of brazilin on serum cytokine and enzyme levels in CIA mice

**Figure 7:**
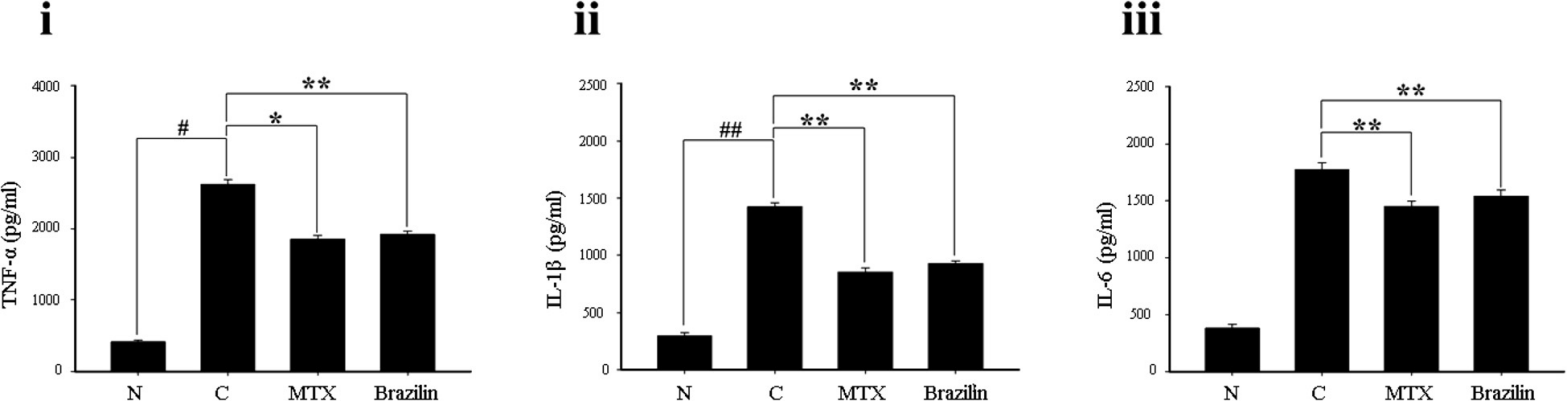
Effects of brazilin from *Caesalpinia sappan* L. on serum tumor necrosis factor (TNF-α) **(i)**, interleukin (IL)-1β **(ii)**, and IL-6 **(iii)**, levels. On day 42 post-administration, the levels of inflammatory cytokines in mouse sera were determined via enzyme-linked immunosorbent assays. Data are expressed as means ± standard errors; *n* = 10 in each group. **p < 0.001*,* *p < 0.05, versus control group mice with CIA; ^##^ *p <* 0.001, ^#^ *p <* 0.05, versus normal mice.

The serum levels of the proinflammatory cytokines TNF-α, IL-1β, and IL-6 in CIA-induced mice are shown in Figure 7. Serum cytokine levels in the CIA-induced control group were significantly higher than those in the normal group on day 42. Significant reductions in the cytokine levels were evident upon treatment with either MTX or brazilin, compared to the control group. Although the levels of TNF-α, IL-1β, and IL-6 in serum were not significantly different between MTX or brazilin administered groups, we observed reduced-level of serum cytokines in these groups when compared with control group.

### Effects of brazilin on organ weights and biochemical parameters in CIA mice

Brazilin caused marginal weight loss in CIA-induced mice compared to normal animals, but this was not accompanied by any observable toxicity. Relative organ weight means that the organ weight is based on proportionality. In such cases, organ weights are normalized by body weight, thereby permitting comparison of organs from animals with different body weights. The liver-to-body weight ratios of control group against normal group was 6.01 ± 0.48, whereas MTX and brazilin administrated groups were 4.84 ± 0.62, and 6.03 ± 0.72, respectively. The kidney-to-body-weight ratio in control group was 1.98 ± 0.11, while MTX and brazilin were 1.63 ± 0.11 and 1.92 ± 0.13, respectively. In brazilin-administered group, no significant changes were found in liver, kidney, and spleen organ-to-body weights (Table 2). Serum AST levels were higher in the control group than the normal group, and brazilin caused a marginal reduction in AST levels. Higher ALT levels were evident in the CIA-induced control group compared to normal group. Administration of brazilin to CIA-induced mice caused the ALT level to be maintained at a level greater than the control group. Mice given MTX did not show an appreciable rise in ALT levels, compared to brazilin-treated animals. ALP levels were also higher in CIA-induced mice (compared to normal and control) after treatment with either MTX or brazilin. Thus, brazilin, a natural component of the heartwood extract of *C. sappan,* effectively regulated stress levels after induction of type-II CIA.

**Table 2:** **Comparison of relative organ weights and biochemical parameters in DBA/1J mice with CIA**

**Groups (** ***n*** **= 10)**		**Normal**	**Control**	**MTX**	**Brazilin**
**Average relative organ weights (organ weight/body weight, %)**					
	**Liver**	4.93 ± 0.10	6.01 ± 0.48*	4.84 ± 0.62	6.03 ± 0.72
	**Kidney**	1.98 ± 0.11	1.95 ± 0.32	1.63 ± 0.11*	1.92 ± 0.13
	**Spleen**	0.25 ± 0.02	0.48 ± 0.14*	0.34 ± 0.10	0.56 ± 0.21
**Biochemical parameters**					
	**AST(U/L)**	127.50 ± 29.10	172.50 ± 30.51	142.23 ± 30.17	145.00 ± 32.71
	**ALT(U/L)**	32.00 ± 2.83	44.50 ± 3.54*	43.33 ± 12.58	37.50 ± 3.54*
	**Creatinine (mg/dL)**	0.53 ± 0.11	0.70 ± 0.11	0.55 ± 0.05	0.55 ± 0.09

Organ coefficients (liver, kidney, and spleen) for DBA/1J mice treated with saline, MTX (3 mg/kg of body weight; injected every third day from day 22 to day 42 after 2nd booster injection) or brazilin (10 mg/kg of body weight; injected daily from day 22 to day 42 after the 2nd booster injection). Statistical analysis was performed with a 2-sample t-test comparing each sample group to the related normal group. Note: *denotes statistical significance at *p <* 0.05 versus normal group.

## Discussion

*C. sappan* (Leguminosae) is an herb exhibiting ethnomedical properties [22]. The herb is distributed in Southeast Asian countries, including China, India, Burma, Thailand, Indonesia, and Vietnam. The heartwood of the plant has traditionally been used as an analgesic [23], although antioxidative [24], anti-inflammatory [25-27], antibacterial [28,29], and anticonvulsive [25] activities have also been reported. *C. sappan* extracts have been shown to exert an anti-osteoporotic activity, and also anti-inflammatory actions on osteoarthritic chondrocytes and synovial macrophages [30,31]. The plant is a rich resource of flavonoids [32] and phenolics including prostosappanins A-E, sappanchalcone, 3-deoxysappanone, 7, 3′, 4′-trihydroxy-3-benzyl-2H-chromene, and others [33,34]. Brazilin exhibits various biological activities including anti-hyperglycemic [15], anti-hepatotoxic [14], and anti-inflammatory effects [17]. Earlier reports also suggested that, *in vitro,* brazilin decreased the levels of mRNAs encoding proinflammatory cytokines such as TNF-α and IL-6 [35]. Indeed, studies have shown that immune cell lines, including RAW 264.7 mouse macrophages, mouse macrophage-like J774.1 cells and THP-1 cells, produce proinflammatory cytokines, chemokines, and other proinflammatory mediators results in an inflammatory environment that drives the upregulation of cartilage-degrading matrix metalloproteinases (MMPs), disintegrin, and metalloproteinase with thrombospondin motifs (ADAMTS) [36]. It was reported that brazilin obtained from an ethanolic *Caesalpinia sappan* extract (CSE) inhibited the expression of proinflammatory cytokines IL-1β and TNF-α in IL-1β-stimulated chondrocytes and LPS-stimulated THP-1 macrophages [30].

Brazilin was the principal component of the most active fraction obtained after sequential purification of *C. sappan* extracts via Sephadex and silica gel chromatography, and yielded a near-homogenous HPLC peak. Subsequently, the structure thereof was elucidated via MS and NMR spectroscopy. In a previous study, nine compounds from *C. sappan* purified by silica gel chromatography included brazilin and sappanchalcone, protosappanin, 3-(3′, 4′-dihydroxybenzyl)-7-hydroxy chroman-4-one, episappanol, 4-O-methylepisappanol, 4-O-methylsappanol, 3-deoxysappanchalcone, and 4-(7-Hydroxy-2,2-dimethyl-9βH-1,3,5-trioxa-cyclopenta[α]naphthalene-3α-yl-methyl)-benzene-1,2-diol. Brazilin was earlier isolated from an ethyl acetate extract of *C. sappan* using preparative HPLC with application of 100% methanol for 45 min [30], similar to our purification method. HPLC separations of methanol and ethanol extracts of *C. sappan* yielded peaks similar to those obtained upon HPLC of the n-hexane extract. All extracts stimulated osteoblast proliferation and exhibited anti-osteoporosis activities [31]. The spectral features of brazilin isolated from ethyl acetate extracts of *C. sappan* in the present study were in agreement with spectral profiles reported in the literature [25,37].

We studied the *in vivo* anti-inflammatory effects of brazilin in a collagen-induced arthritis (CIA) mice model. CIA mouse model can be used to explore human RA, because humans and mice are similar both genetically and immunologically [38]. CIA has been widely used as the animal model in RA research and demonstrated to resemble human RA more closely in terms of clinical, histological and immunological features, as well as genetic linkage, compared with other experimental arthritis models [39].

In a preliminary study, brazilin isolated from *Caesalpinia sappan* L. (Leguminosae) was implicated as a potent NF-ĸB inhibitor that selectively disrupts the formation of the upstream IL-1R signalling complex. Analysis of upstream signalling events revealed that brazilin markedly abolished the IL-1β-induced polyubiquitination of IRAK1 and its interaction with IKK-G counterpart. Notably, pre-treatment of brazilin drastically interfered with the recruitment of the receptor-proximal signalling components including IRAK1/4 and TRAF6 onto MyD88 in IL-1R-triggerd NF-ĸB activation. Interestingly, brazilin did not affect the TNF-induced RIP1 ubiquitination and the recruitment of RIP1 and TRAF2 to TNFR1, suggesting that brazilin is effective in selectively suppressing the proximal signalling complex formation of IL-1R, but not that of TNFR1 [40].

We induced the development of chronic inflammation and closely monitored the effects thereof in terms of joint edema, erythema, and immobilization. The mean arthritis index score was 2.5-3.5 prior to administration of MTX and/or brazilin. MTX, a DMARD, is commonly used to reduce inflammation and joint destruction in RA patients, but continuous prescription thereof may cause cytotoxic and genotoxic complications [41,42]. Our data shows that brazilin inhibits a higher RA disease progression in mice as compared to anti-rheumatic drug MTX. The serum levels of inflammatory mediators decreased after administration of brazilin in the present study that is in agreement to the several reports on attenuation of rat CIA by heartwood extracts of *C. sappan* [43].

Wang *et al.* reported that the ethanol extracts of *C. sappan* reduced the serum levels of proinflammatory cytokines including IL-1β, IL-6, and TNF-α and expression of the COX-2 and NF-ĸB transcription factors in paw cartilage induced in CIA rat (Wistar strain) model [39]. The incidence of arthritis in CIA-susceptible strains of mice is generally very high, reaching 50–100% in most strains [44]. The arthritis incidence rate in male Wistar rat model was 10-75% [39,43], lower than 80-100% in the DBA/1J mouse model used in present study.

To evaluate adverse effects of brazilin, we physiologically monitored the arthritis disease development in mice in which CIA had been induced (control group), and in animals receiving MTX and/or brazilin, to assess whether the drugs might inhibit disease progression. We found that intraperitoneal administration of 10 mg/kg brazilin reduced paw swelling to a level comparable to that noted upon MTX treatment at a dose of 3 mg/kg body weight every 3rd day.

BMD volumes in CIA-induced control group were significantly lower, compared to test volumes, in the left tibial metaphysis, the distal part of the calcaneous, and the distal part of the metatarsal bone, but did not improve after administration of MTX or brazilin. The BMD decreases in control group may be attributable to increased bone surface erosion and decreased trabecular thickness. However, both bone volume and surface area were greater in the control group. These results agree with those of a study on BMD and micro-CT joint projection after administration of bisphosphonate, minodronic acid [45]. High-level bone turnover rate near joints was also observed in a model of carrageenan-induced arthritis [46]. Both suppression and reduction of BMD loss near joints are measures of anti-arthritic efficacy [47,48]. Suppression of bone surface erosion, and maintenance of trabecular thickness and the trabecular bone pattern, upon brazilin administration, reduced the extent of damage to both bone and joints. Therefore, brazilin from *C. sappan* maintained BMD and bone microstructure, without inhibiting mineralization.

## Conclusions

We found that brazilin prepared from ethyl acetate extracts of *C. sappan* effectively reduced the serum levels of TNF-α, IL-1β, and IL-6; maintained the bone surface pattern; and decreased both paw swelling and the development of gross lesions, in the CIA mice model. These results suggest that brazilin derived from the heartwood of *C. sappan* may be useful to treat both RA and other inflammatory disorders.

## Abbreviations

CIA: Collagen-induced arthritis
MTX: Methotrexate
RA: Rheumatoid arthritis
TNFα: Tumor necrosis factor α
IL: Interleukin
NSAIDs: Non-steroidal anti-inflammatory drugs
DMARDs: Disease modifying anti-rheumatic drug
LC-MS-IT-TOF: Liquid chromatography-mass spectrometer inductively coupled-time of flight
NMR: Nuclear magnetic resonance
CFA: Complete freund adjuvant
ICFA: Incomplete freund adjuvant
BMD: Bone mineral density
AST: Aspartate transaminase
ALT: Alanine transaminase
ALP: Alkaline phosphatase
Fr: Fraction
